# Reward prediction error in learning-related behaviors

**DOI:** 10.3389/fnins.2023.1171612

**Published:** 2023-08-17

**Authors:** Yujun Deng, Da Song, Junjun Ni, Hong Qing, Zhenzhen Quan

**Affiliations:** ^1^Key Laboratory of Molecular Medicine and Biotherapy, School of Life Science, Beijing Institute of Technology, Beijing, China; ^2^Department of Biology, Shenzhen MSU-BIT University, Shenzhen, China

**Keywords:** reward prediction error, dopamine, associative learning, reversal learning, reinforcement learning

## Abstract

Learning is a complex process, during which our opinions and decisions are easily changed due to unexpected information. But the neural mechanism underlying revision and correction during the learning process remains unclear. For decades, prediction error has been regarded as the core of changes to perception in learning, even driving the learning progress. In this article, we reviewed the concept of reward prediction error, and the encoding mechanism of dopaminergic neurons and the related neural circuities. We also discussed the relationship between reward prediction error and learning-related behaviors, including reversal learning. We then demonstrated the evidence of reward prediction error signals in several neurological diseases, including Parkinson’s disease and addiction. These observations may help to better understand the regulatory mechanism of reward prediction error in learning-related behaviors.

## 1. Introduction

Learning plays a key role in response to diverse stimuli and decision-making in all animals. Animals need learn to predict the outcomes of different actions, to associate and compare the likelihood of future events and, ultimately, decide accordingly. In doing so, animals are constantly shaping their expectations and actions to the variegated external environments guiding by the prediction error ability (Schultz and Dickinson, 2000; Schultz, 2016a). Prediction error represents a mismatch between reality and prediction. In learning process, prediction error is proceeded if and only if such a discrepancy occurs (Rescorla and Wagner, 1972a). When this discrepancy is caused by reward or absence of reward, prediction error will be called as positive or negative reward prediction error, which has been revealed to involve in many learning processes.

**Figure 1:**
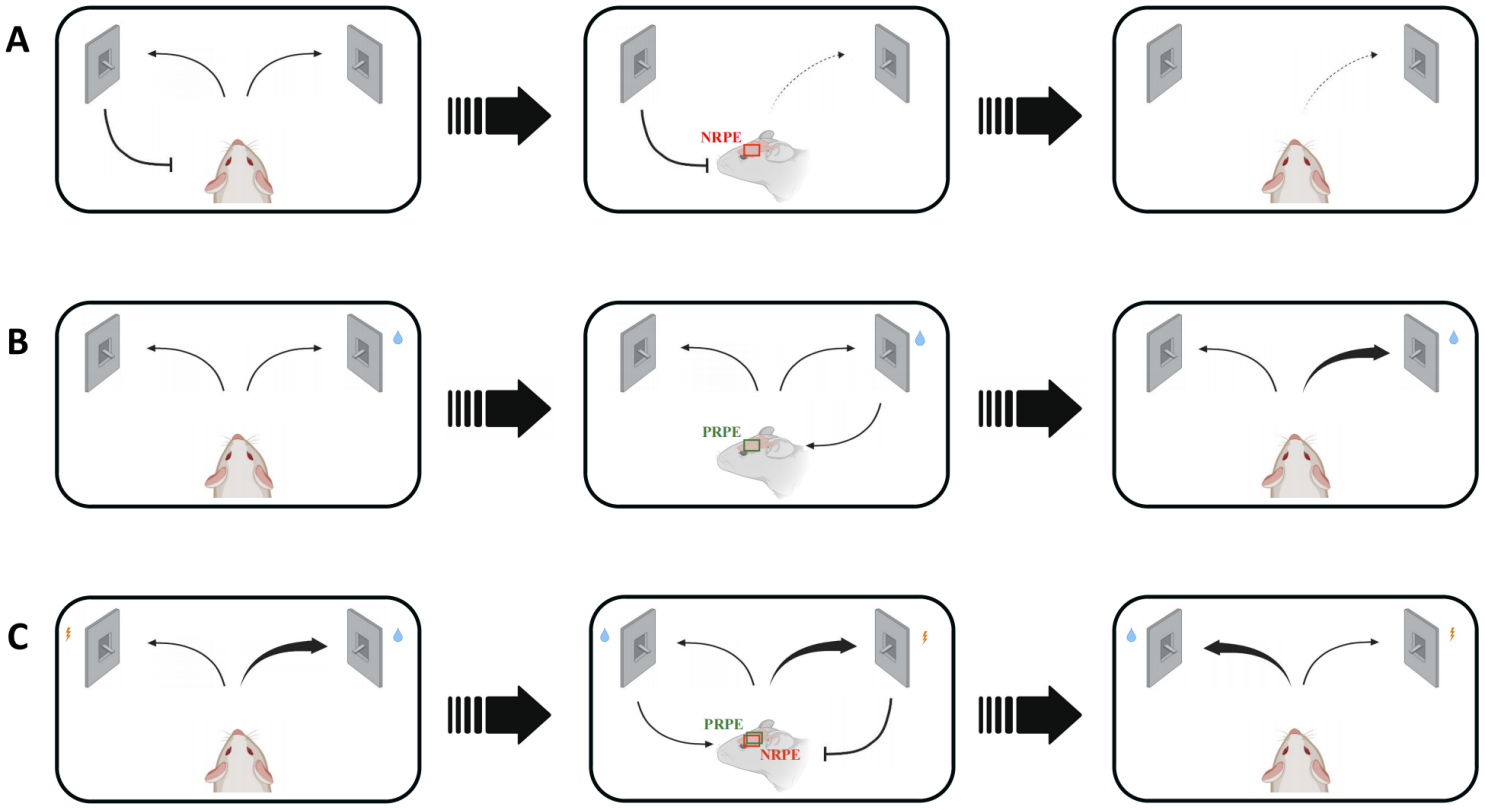
Various situations and consequences of mice regulated by reward prediction error signals in reward-based learning process. NRPE: negative reward prediction error, PRPE: positive reward prediction error. **(A)** The wrong response will lead to NRPE, which would result in less choice of false response to avoid similar negative outcomes in the future. Red box shows the NRPE signal in mice brain. **(B)** The right choice will bring PRPE, which would result in more choice of the right response. Green box shows the PRPE signal in mice brain. Reinforcement learning involves in the utilization of both positive and negative RPE. **(C)** The reversal learning could be affected by both positive and negative RPE, during which the response-outcome relevance is easily to change. Red and green box shows both NRPE and PRPE signals in mice brain.

In associative learning process, an animal needs to learn the cue-response relationship and adjust its behavioral choice by the guidance of reward prediction error signal. In addition, reversal learning and reinforcement learning also represent the ever-changing and consistent environments, which require the effect of reward prediction error to correct an animal’s response after receiving an unconditional stimulus (Figure 1). These behavioral protocols allow us to explore the regulatory role of reward prediction error with different learning processes in multiple perspectives.

Dopamine is a well-explored modulatory neurotransmitter. There are abundant researches providing insights into the functions of dopamine, including learning, reward, motivation and so on (Diederen and Fletcher, 2021). Researchers believed that dopamine represents the degree of “pleasure” or “happiness” in prediction, anticipation and reward-seeking behaviors. Though it has been evidenced that the dopaminergic neurons perform their role in signaling the absence of reward (Friston, 2010), most researches still focus on the relationships among the reward, behavior and dopamine signals, which includes the reward prediction error signal (Schultz, 2016b).

In this review, we first narrate the concept of reward prediction error to better understand the learning process, and discussed the dopamine signaling in the encoding of reward prediction error in detail. We then review the model of associative learning and the memory reconsolidation related with the reward prediction error signal. As a case in point, we elaborate the concept, neuron mechanism and hypothesis about reversal learning and reinforcement learning. Lastly, we indicate the reward prediction error in the Parkinson’s disease and addiction, and provide an overview of a recent study about the reward prediction error signal.

## 2. Reward prediction error

### 2.1. Concept of reward prediction error

Humans have long been curious about how our brain understands the world, and assess a current situation, and commands movement by afferent information from sensory inputs to earn a reward and avoid danger. In the theory of neuroscience, predictive coding remains the most influential that the brain is predicting continuously during sensing, learning or decision-making. In this process, the brain models the world according to differences between predicted and actual conditions. This deviation is termed prediction error, which is the most significant concept in predictive coding. During the learning progress, reward prediction error plays a crucial role in decision-making. Reward prediction error refers to differences between expected and actual rewards. “Reward” represents any object, event, stimulus, situation or activity that can promote positive learning, induce approach behavior, maximize decision-making or trigger positive emotions (Schultz, 2017).

Reward prediction error can be positive or negative, depending on whether the predicted reward value surpasses the actual value, which is the signed reward prediction error (Montague et al., 1996). When the actual reward value surpasses the predicted value, the reward prediction error is positive, which could enhance the attention on the reward related cues (Mackintosh, 1975). Conversely, when the predicted value surpasses the actual reward value, the reward prediction error is negative. The positive prediction error can promote learning or behavioral responses (Schultz et al., 1997; Schultz, 2017; Ergo et al., 2020). In contrast, the negative reward prediction error could promote learning to avoid an analogous condition (Schultz et al., 1997; Schultz, 2017; Rolls, 2019; Starita et al., 2019). Both positive and negative reward prediction error signal can drive learning (Pearce and Hall, 1980), such as, reinforcement learning and reversal learning processes (Rescorla and Wagner, 1972a; Fouragnan et al., 2017).

### 2.2. Dopaminergic neurons encode reward prediction error signal

The relationship between reward prediction error and the activity of dopaminergic neurons was first reported by Schultz et al. (1997). In the last 20 years, technological breakthroughs, including optogenetics, have considerably advanced research on dopaminergic neuron function. Steinberg et al. (2013) determined the role of dopaminergic neurons in the reward prediction error hypothesis. In a behavioral procedure known as “blocking,” animals were required to learn the relationship between cue A and reward and subsequently between cue AB and reward. Further experiments on the same batch of animals revealed that they learned nothing about cue B and reward. Since cue A can perfectly forecast the reward, there is no reward prediction error in the presentation of cue B. But optogenetically stimulating ventral tegmental area (VTA) dopaminergic neurons unblocked learning. Maes et al. (2020) used a similar procedure involving optogenetic inhibition of VTA dopaminergic neurons, which showed that these neurons encode the error between prediction and reality, not reward predictions. Together, recent studies in dopamine have substantially complemented the reward prediction error hypothesis, underscoring the importance of dopaminergic neurons for this hypothesis (Diederen and Fletcher, 2021; Lerner et al., 2021; Farrell et al., 2022).

**Figure 2:**
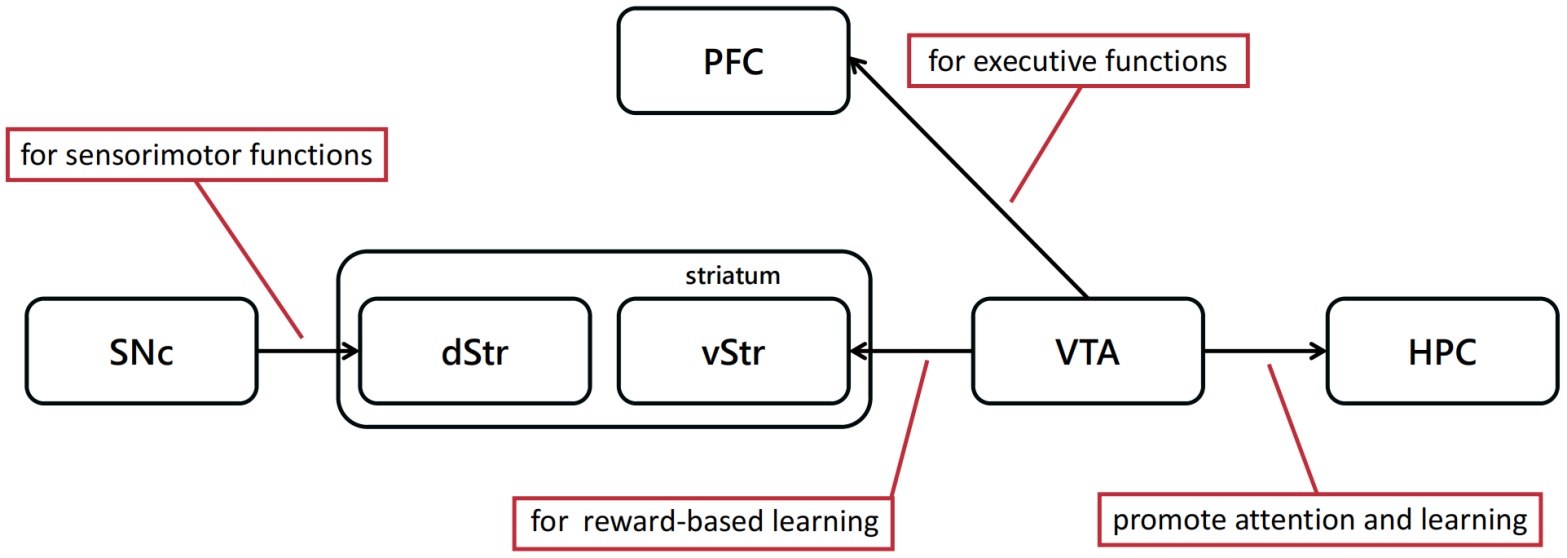
Dopaminergic neural pathways play a critical role in cognitive behavioral experiment protocols discussed above. The dopamine projections from SNc to dorsal striatum(dStr) are mainly associated with sensorimotor functions, and from VTA to ventral striatum(vStr) primarily associated with limbic-related functions.

Review by Schultz (2007) expounded the function of dopaminergic neurons in movement, learning, attention, reward, punishment and so on (Figure 2). Besides, they sorted the research about the reward signal in electrophysiology, and its relationship with reward prediction error. When animals perceive the reward, these neurons respond to process information, such as reward quantity, probability, risk, subjective value or utility, among other variables. Most dopaminergic neurons in the substantia nigra pars compacta (SNc) and VTA produce a brief, phasic response soon after perceiving the reward. This signal reflects the difference between a received and a predicted reward (Waelti et al., 2001; Tobler et al., 2003; Bayer and Glimcher, 2005; Pan et al., 2005; Lak et al., 2014; Ergo et al., 2020), which is extremely different from the slower dopamine activity (Fiorillo et al., 2003). Recent research has demonstrated that three types of signals are encoded by dopaminergic neurons (Schultz, 2016a). One is a consistent signal, which may be unrelated to reward prediction error since it has no relationship with time lapse or given reward, but possibly influence the function of movement, cognition and motivation (Schultz, 2007). The other two are both stimulus-related signals. Fast signals occur hundreds of milliseconds after the stimulus perception and disappear quickly; slow signals peak around 10 min after the stimulus perception (Nomoto et al., 2010).

The sub-second-fast dopaminergic signals provide information about reward prediction error, whereas slow signals contain information about movement, cognition, attention and motivation. Schultz (2007) reported that these sub-second signals can be measured by electrophysiology or voltammetry, and described its relationship with reward prediction error. The signals can be divided by statistics into two parts (Schultz, 2016a). The first part represents the response to the emerging reward, such as perceiving the presence and recognizing the type of reward. The second part encodes the subjective value of reward. Evidence from behavioral experiments shows that this value-encoding signal is weakened by temporal discounting and aversive stimuli (Schultz, 2017). Although this detection-discrimination hierarchical processing also occurs in other neurons (Thompson et al., 1996), only dopaminergic neurons in the midbrain dopamine system determine the strength of reward prediction error.

Besides, dopamine receptors could have different functions in reward prediction error signal transmission. In striatum, D1 receptors are mainly localized in the neurons projecting to pallidum and substantia nigra pars reticulata, whereas D2 receptors are mainly localized in the neurons projecting to external pallidum (Hersch et al., 1995). Of all D1 receptor, 80% are in low-affinity state, the other 20% are in high-affinity state (Richfield et al., 1989). The D2 receptors differ from D1 receptors for about 80–90% of D2 receptors are in high-affinity state, whereas only 0–10% are in low-affinity state. Taken together, D1 receptors have nearly 100 times lower affinities than D2 receptors have (Schultz, 1998). The reward prediction error encoded by dopaminergic neurons could have different influence for the difference in the location and affinity of dopamine receptors. The dopamine release caused by reward or reward prediction could influence D1 and D2 receptors in striatum. But the reduction of dopamine release caused by the reduction or deletion of reward would reduce the stimulation of D2 receptors for its higher affinity. Thus positive reward prediction error signal would have influences on most of striatal dopaminergic output neurons, whereas the negative reward prediction error signal mainly influence the neurons projecting to external pallidum (Schultz, 1998).

The reward prediction error can be positive or negative, depending on whether the actual reward is bigger than prediction. The positive and negative reward prediction error signals can be widely found in lateral habenula neurons, or the specific neurons in striatum, globus pallidus, amygdala, anterior cingulate cortex and supplementary eye field (Bermudez and Schultz, 2010; So and Stuphorn, 2012; Schroll et al., 2015; Schultz, 2017; Alexander and Brown, 2019; Lee and Hikosaka, 2022; Basanisi et al., 2023).

Dopaminergic neurons in VTA can induce conditioned place preference (Tsai et al., 2009), which indicates better context associations based on reward (McKendrick and Graziane, 2020). In turn, GABAergic neuron inhibition interferes with reward-based behavior (Van Zessen et al., 2012). These mechanisms coincide with the ability of VTA dopaminergic neurons to encode reward value and are regulated by GABAergic neurons. Simultaneously, different brain regions in reward-related pathways perform different functions. For example, glutamatergic neurons projecting from the basolateral amygdala to the nucleus accumbens encode reward behaviors (Stuber et al., 2011), whereas glutamatergic projections from the basal ganglia to the ventral tegmental area encode aversive behaviors, and GABAergic projections encode positive behaviors (Jennings et al., 2013). Therefore, in different regions, dopaminergic neurons may have different molecular signatures supporting their specific functions.

## 3. Reward prediction error and behavior

Several studies have reported that the reward prediction error signal coded by dopaminergic neurons is necessary for cue-reward association learning and consolidation via activating or inhibiting dopaminergic neurons (Steinberg et al., 2013; van Zessen et al., 2021; Nishioka et al., 2023). Also, Reward prediction error is tightly correlated to other learning-related behaviors, such as reversal learning and reinforcement learning (Rescorla and Wagner, 1972a; Fouragnan et al., 2017; Katthagen et al., 2020). We thus illustrated the relationships of reward prediction error with several types of learning processes in this section.

### 3.1. Research models in associative learning

Associative learning refers to the process of acquiring associations between different environmental events that occur in close temporal or spatial proximity, or when one event reliably predicts the occurrence of another (Takehara-Nishiuchi, 2022). Researchers have studied the role of reward prediction error in associative learning for a long time. In the last century, Rescorla and Wanger proposed a model whereby synaptic strength becomes stronger when reward is more valuable than prediction and weaker otherwise (Rescorla and Wagner, 1972a). The model indicates that the learning process depends on the prediction error. Building on the Rescorla-Wagner model, temporal difference learning was subsequently proposed as an improvement to the previous model (Sutton and Barto, 1987; Sutton and Barto, 1998). In temporal difference learning, the prediction error is the difference between the expected value of all future rewards at a specific point in time and at later time points. Some studies on ventral tegmental area (VTA) highlighting the activity pattern of dopaminergic neuron in this area have supported this model (Eshel et al., 2016). Studies in primates have also indicated that this model can be used to predict expected reward in gambling (Stauffer et al., 2014). Furthermore, this model was also confirmed in studies about artificial intelligence. For example, the algorithm based on temporal difference learning can be used to solve challenging tasks which traditional artificial intelligence cannot (Mnih et al., 2015). Broadly speaking, reward prediction error is crucial for understanding the learning process physiologically and behaviorally.

### 3.2. Memory reconsolidation during learning

For decades, studies involving humans and other animals have demonstrated that reward prediction error, or “mistake,” is crucial for promoting memory change (Schultz et al., 1997; Schultz, 2017; Sinclair and Barense, 2019). In the learning process, learners must adjust their own strategy in a timely manner to the conditional response to maximize the reward or minimize the loss. Learning driven by negative response primarily consists of updating memory adaptively when the learner encounters information that contradicts prior experiences. But how does this ‘error’ renew cognition and thus change behavior? According to the memory reconsolidation theory, memory reconsolidation reactivates and temporarily destroys established long-term memories (Miller and Springer, 1973; Lewis, 1979; Lee, 2009; Lee et al., 2017; Sinclair and Barense, 2019). After several hours of protein synthesis, memory will be restabilized and consolidated. The reconsolidation process is usually divided into three parts, namely encoding, reactivation and detection (Sinclair and Barense, 2019). Numerous studies have interfered with this process in different ways, all of which have found that memory is suppressed or distorted after the reactivation process (Das et al., 2018).

In the learning process, prediction error is a prerequisite for memory reconsolidation (Krawczyk et al., 2017). For example, in the Pavlovian conditioning experiment, after pairing conditioned and unconditioned stimuli, giving the conditioned stimulus alone in the experiment will also cause prediction error. This incomplete cue will promote the reconsolidation process of human memory (Sinclair and Barense, 2018). In another study about aversive associative memory, providing incomplete cues positively affects visual fear memory (Schiller et al., 2010).

In addition to associative learning reconsolidation, incomplete cues also trigger changes in other types of memory, such as complex episodic memory. For example, in one study, subjects were shown a series of videos with a strong narrative, and the next day some videos were played and stopped before the outcome, thereby producing prediction error. This study showed that the subjects who had observed interrupting videos are more likely to generate false memories (Sinclair and Barense, 2019). Similar experiments have been performed in rodents (Krawczyk et al., 2017) and humans (Sevenster et al., 2013). In short, such incomplete and unexpected cues both disrupt and update the original memory.

Similar incomplete cues, however, cannot lead to memory updating. For example, when museum visitors were shown pictures and distraction pictures during their visit, which may be regarded as incomplete cues for the tour process, whether in chronological order or not, the cues that disrupted the chronological order reduced the accuracy of location recall and the process of memory updating (St Jacques and Schacter, 2013). Accordingly, prediction errors, which represented by incomplete cues, may lead to memory updating in most cases, underlying error-driven learning processes.

Though the unsigned reward prediction error can represent how ‘surprised’ subjects were with these ‘incomplete cues’, we are not yet able to quantify this process. Furthermore, it is still unknown whether memory changes are due to reward prediction error or interference from retrospective inhibition. Some studies have attempted to use real-time functional magnetic resonance imaging (fMRI) to detect changes in neural activity during the retrieval phase of episodic memory in subjects (DeBettencourt et al., 2019). However, further research is needed to analyze the mechanisms of regulation of reward prediction error and learning reconsolidation.

### 3.3. Reward prediction error and reversal learning

Reward prediction error occurs in many learning-related behaviors. In the sections below, we studied the function of reward prediction signal in behavioral changes in two conditions. One is the reversal learning, which represents the learning process in the ever-changing environment. The other is the reinforcement learning, which represents the organism repeats one specific behavior which will bring positive outcome during the operant learning process in the constant condition.

#### 3.3.1. The concept of reversal learning

Reversal learning refers to a set of paradigms used for assessing cognitive flexibility by evaluating adaptive responses in the changing stimulus-outcome or response-outcome contingencies (Izquierdo et al., 2017). For example, the common visual reversal learning task has at least two types of visual stimuli for the animal to learn in experiments. Some choices lead to reward, whereas others lead to punishment. For both types of stimuli condition, the results associated with the stimuli will be exchanged after a specific number of experiments. The stimulus previously associated with the reward will lead to the punishment, and the stimulus previously associated with the punishment will in turn lead to the reward. During the whole process, this exchange can be repeated many times. Throughout the experiment, the animals will break the original stimulus-result connection multiple times with reversal, and form a new connection. This learning process can be divided into two stages (Swainson et al., 2000): the acquisition stage and the reversal stage. In the acquisition stage, experimental animals mainly complete preliminary learning by associating a stimulus with the corresponding outcome. After reversal, the choice which is related with positive outcomes does not bring reward anymore. Therefore, this kind of choice would bring the negative reward prediction error as the actual reward is much lower than the predicted reward. For the same reason, the new reward-related choice would bring positive reward prediction error, for it provides actual reward with no prediction reward. During the reversal stage, the learning criterion is achieved by updating the original stimulus-outcome relationship. As the experiment progresses, the animal will become more familiar with the procedure and spend less time in the reversal stage. In complex and uncertain environment, the ability of reversal learning is particularly important for helping organisms behave adaptively to earn more feedback, or avoid punishment.

#### 3.3.2. Neural mechanisms of reversal learning

Butter (1969) conducted the first reversal learning experiment. At the beginning of the experiment, two visual stimuli were given to macaques, and one of the stimuli was bound to a reward. Once the macaques learned to choose the correct image for the reward, the reward was paired with another visual stimulus. Normal macaques adjusted their choices quickly when the reward was reversed, and increasing the number of reversals accelerated the correction of their choices. In contrast, macaques with completely destroyed orbitofrontal lobes took much longer to learn new choice after reversal, and the learning rate hardly increased with the number of reversals. Based on these results, some studies have proposed a response theory. In the response theory, the orbitofrontal cortex plays a key role in inhibiting the original choice and weakening the original stimulus–response connection. But another theory was also proposed, termed value theory. According to the value theory, the orbitofrontal cortex is responsible for encoding either two visual stimuli whereby the subjective value is continuously updated with feedback during the learning period or the strength of the stimulus–reward connection, which it is adjusted over time.

Based on the two hypotheses above, many studies have analyzed the role of brain regions such as the orbitofrontal cortex in the process of reversal learning. The results showed that the nerve fibers in the orbitofrontal cortex are essential in reversal learning, not the neurons in the orbitofrontal cortex. These nerve fibers update the subjective value of the stimulus at any time (Rudebeck et al., 2013). The orbitofrontal cortex and the amygdala have functions in reward or punishment-related reversal learning tasks and different ‘learning speeds’. The orbitofrontal cortex affects learning flexibility by affecting the encoding of stimulus–response connections in the basolateral amygdala (Stalnaker et al., 2007; Morrison et al., 2011). Furthermore, GABAergic neurons in the orbitofrontal cortex projecting to the striatum are crucial for reversing the original stimulus-result connection that inhibits learning (Yang et al., 2021). In conclusion, distinct orbitofrontal cortex-amygdala-striatal circuits mediate different parts of the reversal learning and subsequent decision-making process (Groman et al., 2019).

In addition, many previous physiological, pathological and imaging studies have indicated that the frontal cortex and amygdala are important for reward, punishment and related decision-making processes (O’Doherty et al., 2001; Baxter and Murray, 2002; Holland and Gallagher, 2004). The frontal cortex also has bidirectional projections with amygdala (Cavada et al., 2000). Given the characteristics of the reversal learning task, these two brain regions likely play a key role in this learning process (Trinh et al., 2012). On the one hand, the human amygdala is crucial for representing expected rewards in the frontal cortex, which can guide future behavior (Hampton et al., 2007). On the other hand, demonstrating that the midbrain dopamine system, which encodes reward value, plays a key role in reversal learning, would provide the strongest evidence for the value theory. Accordingly, studies have shown that mice lacking TGF-β signaling in midbrain dopaminergic neurons are significantly impaired in establishing new stimulus–response connections during reversal learning (Luo et al., 2016). Moreover, dopamine signaling in both the striatum and amygdala is essential in the reversal learning task (Costa et al., 2016).

Regardless of reversal-learning hypothesis, at this process covers many advanced cognitive functions of the brain, including learning simple conditioned reflexes, predicting future rewards, recognizing missing rewards, and changing to previous perceptions and behavior in similar circumstances. Since so many brain regions and functions are involved in these cognitive and behavioral functions, reversal learning can be analyzed in different brain regions. Neurons in the anterior cingulate cortex have the ability to integrate outcomes with actions, thereby drawing on past experiences to guide future behavior (Shima and Tanji, 1998; Williams et al., 2004). Therefore, neurons in the anterior cingulate cortex area are crucial for reversal learning tasks requiring adjusting behaviors over time according to environmental changes (Kawai et al., 2015). The lateral habenula, where most neurons are activated by reward omission, aversive cues, and predictions (Matsumoto and Hikosaka, 2007; Matsumoto and Hikosaka, 2009), has a similar function. Therefore, studies have also confirmed that lateral habenula plays a key role in reversal learning tasks (Kawai et al., 2015).

In the reversal learning task, the process between two reversals can be regarded as a reinforcement learning process. The midbrain dopaminergic neurons encode reward prediction error signals, thereby driving plasticity in the striatum to facilitate reinforcement learning (Schultz, 2015). Thus, reversal learning tasks are often analyzed in research on reinforcement learning. For example, a study by Costa et al. (2016) on the contribution of the amygdala and ventral striatum to reinforcement learning mentioned the function of these two brain regions to reinforcement learning in uncertain environments, that is, reversal learning. Reversal learning is closely related to the cognitive flexibility of individuals, so reversal learning tasks are often used in research on cognition and learning flexibility to study synaptic plasticity in the hippocampus (Davenport et al., 2021) and to gather information on diseases such as frontotemporal dementia (Ahmed et al., 2022).

### 3.4. Reinforcement learning and reward prediction error

#### 3.4.1. Reinforcement prediction error

‘Reinforcement’ refers to the process whereby an animal acquires one specific behavior which will bring about a positive outcome in a specific state and learns to link behavior and outcome (Shibata et al., 2022). During this process, the neutral stimuli are referred to as ‘conditioned reinforcer’. In most cases, predicting and comparing different outcomes of every possible reaction leads to a decision-making behavior, which involves choosing the action with most rewards. The goal of reinforcement learning is to choose actions that maximize rewards and minimize punishments or losses (Neftci and Averbeck, 2019). The reward prediction error guides decision-making during reinforcement learning as the brain compares the predicted and actual reward value and calculates the disparity between them (Garrison et al., 2013). For the same reason, the response learned through reinforcement will tend to extinguish when the reinforcer is no longer paired with reinforced behavioral responses, and this process is known as reinforcement learning extinction (Staddon and Cerutti, 2003; Shibata et al., 2022).

**Figure 3:**
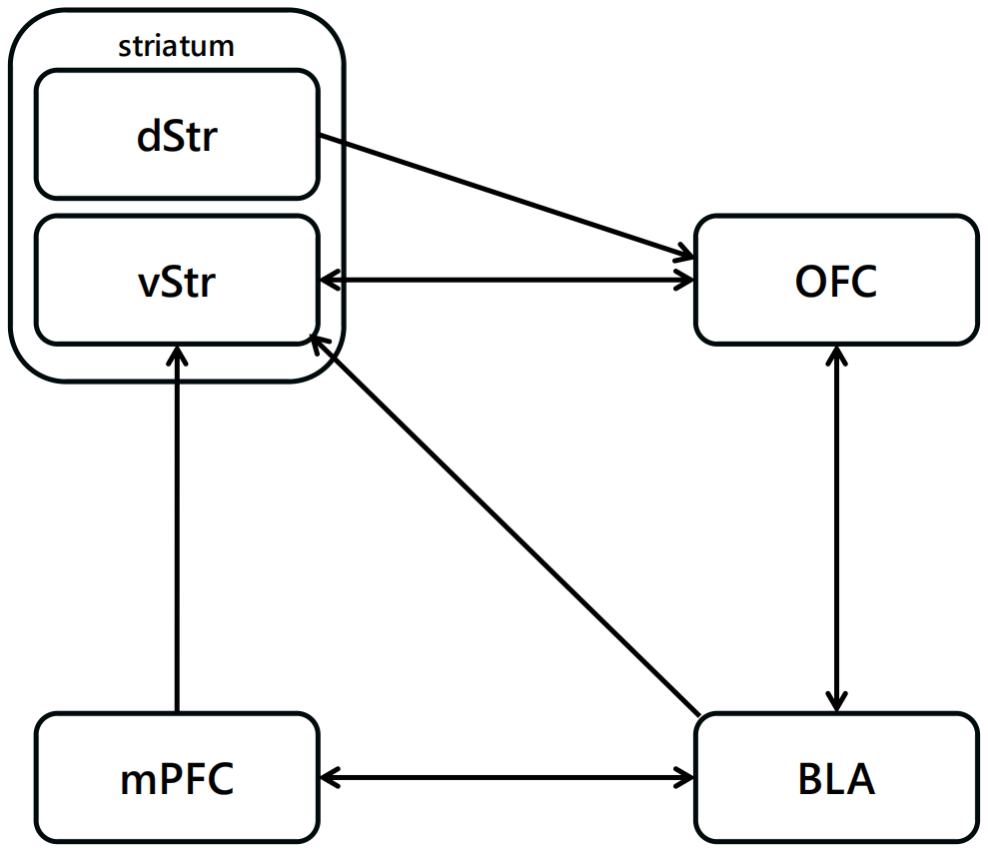
Neural pathways related to reinforcement learning. The projections from BLA to OFC are highly related to the reinforcement with positive outcome, projections from OFC to vStr have function in the reinforcement process with negative outcome.

Edward Thorndike highlighted the essence of reinforcement learning in his study (Thorndike, 1911), stating that “Responses that produce a satisfying effect in a particular situation become more likely to occur again in that situation, and responses that produce a discomforting effect become less likely to occur again in that situation.” The neuronal mechanism of reinforcement learning in mammalian, particularly model-free reinforcement learning, may be one of the most studied systems in neuroscience (Rescorla and Wagner, 1972b; Schultz et al., 1997; Neftci and Averbeck, 2019). The activity of dopaminergic neurons and their activating effects on behavior can be successfully predicted based on temporal-difference reinforcement learning and Rescorla-Wagner theories (Rescorla and Wagner, 1972b). According the model proposed by Jonathan (Mink, 1996), the cortex represents the set of available choices, and cortical synapses on striatal cells encode information about the values of each choice. Activity in striatal cells results in stronger synapses, expressing the values of the options represented by cortex (O’Doherty et al., 2004; Lau and Glimcher, 2008). Striatal activity can be transmitted from the basal ganglia and the thalamus to the cortex or brain-stem motor output areas, resulting in choice behavior (Figure 3). Once the unconditioned stimuli is given, dopaminergic neurons encode a reward prediction error signal (Neftci and Averbeck, 2019).

#### 3.4.2. Reinforcement delay

Several concepts of reinforcement value can be used to summarize the effect of different variables, such as reinforcement delay, reinforcement magnitude, and deprivation level, on behavior (Buriticá and Dos Santos, 2017). Among them, reinforcement delay is the most significant and widely researched concept. In fact, reinforcement delay affects not only the reinforcement learning process but also all mechanisms of associative learning. When I. P. Pavlov. proposed the concept of Pavlovian conditioning forward, reinforcement delay was overlooked. But reinforcement delay soon became a great advance in understanding Pavlovian conditioning. The interval between stimuli is essential in associative learning (Gallistel and Gibbon, 2000), and the interval between a conditioned stimulus and a unconditioned stimulus may also be important for the learning process (Gallistel and Gibbon, 2000).

Broadly speaking, the shorter the period between the last behavior response and the next reinforcer is, the more effective reinforcement will be in modifying such behavior (Jablonsky and Devries, 1972; Miltenberger, 2015). The effect of immediacy on reinforcement learning has been widely documented (Black et al., 1985). A few decades ago, Lea (1979) compared predictions of optimal foraging theory with laboratory research on reinforcement schedules. This experiment showed that animals strongly prefer reinforcement with a shorter delay over a longer delay, even when reinforcement density favors reinforcement with a longer delay. Research has also shown that animals are virtually unable to learn through reinforcement if the reinforcement delay is too long.

Considering the importance of reinforcement delay for associative learning, several studies have aimed at understanding the mechanism of reinforcement learning and temporal relations between events and operant behavior. Most reinforcement delay studies have addressed three research questions (Lattal, 2010). The first question is whether the effect of the interval between responses and reinforcers on reinforcement delay can be separated from its indirect effect on stimuli or reinforcement rate. The second question is whether operant behavior is affected by reinforcement delay or varies with the protocol and condition. The third question refers to the effects of reinforcement delay, which strongly affect the response-reinforcer temporal relation during associative learning and other operating behavioral processes.

The temporal delay between responses and reinforcers is not simply a static parameter for reinforcement learning. Instead, this temporal delay between can have a strong effect on learning process, whether directly or indirectly. Both correlational and mediational accounts of reinforcement delay, in different ways, highlight that disruptions in temporal contiguity determine reinforcement delay effects (Lattal, 2010).

These ongoing research in reversal and reinforcement learning provides deeper insights into the brain and neural systems, fostering outstanding advances in the neural mechanism under the cognition and behavioral change.

## 4. Reward prediction error and diseases

Since encoded by dopamine system, the reward prediction error has been shown its involvement in several neurological diseases, including Parkinson diseases and addiction, that are pathologically related to dopamine system.

### 4.1. Reward prediction error is associated with Parkinson’s disease

Parkinson disease (PD) is a progressive neurodegenerative disorder with many clinical symptoms, such as bradykinesia, rigidity and resting tremor, among others (Lees et al., 2009). The main neuropathological hallmark of PD is dopaminergic neuronal loss in SNc. In PD, the neurodegenerative process begins in the midbrain, especially in these dopaminergic neurons of the substantia nigra. Thus, the clinical changes in motor and cognitive function observed in patients with PD, may help us understand the role of dopaminergic neurons in reward learning and assess the effect of dopamine in reward-based learning underlying the pathological manifestations of basal ganglia, which is crucial for motor function (Alexander et al., 1986), reward and learning (Packard and Knowlton, 2002; Schultz et al., 2003).

The most common treatment for PD is to increase dopamine availability and activity (Van Wouwe et al., 2012) using dopaminergic precursors or dopaminergic agonists. This medication improves the motor function of patients with PD but is less effective in ameliorating cognitive deficits and may even have negative consequences in different cognition functions. For example, reversal learning and extinction learning， which refers to the reduction of the conditioned response as a result of the repeat of conditioning stimulus, can be impaired by dopaminergic medication (Cools et al., 2001). Clinical evidence has also shown that PD patients who receive dopamine treatment develop pathological behaviors, such as gambling, compulsive shopping and eating disorders. These patients may be manifesting hypersensitivity to reward caused by dopamine treatment (Drew et al., 2020). These findings support the “overdose” hypothesis, which explains the negative effect of dopamine medication on some cognitive processes (Swainson et al., 2000).

However, dopamine treatment can still improve the performance of PD patients in some reward-based learning processes. Frank (Frank et al., 2004) showed that dopamine medication helps to learn some actions, but not others. For example, research has indicated that the performance of PD patients in feedback-based learning improves when they are on dopamine medication (Shohamy et al., 2005). These studies on pathological process and treatment of patients with PD shows that dopamine not only plays a key role in reward-based learning but may also have different functions on different types or processes of reward-based learning.

### 4.2. Reward prediction error involves In addiction

Addiction is a type of chronic, recurrent brain disease with extremely complicated pathogenesis, which is often manifested as spontaneous and compulsive behavior (Wise and Robble, 2020). The midbrain dopamine system plays an important role in the forming process of addiction, which highly relies on the dopaminergic projections from VTA to Nucleus accumbens (NAc) (Koob and Volkow, 2016). In drug addiction, after the intake of addictive drugs, the dopaminergic neurons in VTA were activated and encoding the information of the “reward.” Reward prediction error signal in this process were blunted, which makes the pleasant feeling by drug become weaker, resulting in more drug intake to satisfy the drug needed (Lei et al., 2022).

Associative learning is often used in research on the mechanism of addiction curation. For example, some researchers believe that, once a stimulus–response connection is established between addiction elements and feelings of pleasure, a new stimulus–response connection with punishment is difficult to establish and that this difficulty is the essence of addiction (Fernández-Serrano et al., 2012). Studies have reported that substance-related cues can significantly increase dopamine release in the striatum (Everitt and Robbins, 2013), confirming that cocaine, marijuana, and alcohol addicts have impaired reversal learning ability (Pope et al., 2016). fMRI studies have also shown stronger connections between the anterior cingulate cortex and the dorsolateral prefrontal cortex in cocaine users than in normal subjects (Camchong et al., 2011). This result may indicate that addictive elements can enhance existing stimulus–response connections in the brain of patients while interfering with their ability to revise the original connection and form new stimulus–response connections, ultimately manifesting as extreme dependence on addictive substances. From this point of view, research on the mechanism of associative learning is helpful to explore the mechanism of addiction and essential to addiction treatment and prevention.

## 5. Conclusion

Learning process cannot be prosperous every time. When a difference between the expected and actual state of the world is identified, prediction will promote learning, and behavior is corrected accordingly. In the last 50 years, exciting advances have been made as numerous studies have supported the relationship between reward prediction error and learning using various techniques in many species. We have much more understanding about the promotion of dopaminergic neurons to the operant learning process and the dynamic dopamine reward prediction-error signal behind. Nevertheless, many unanswered research questions and challenges lie ahead. Given the complexity of brain, there is much to understand about the concrete neuronal mechanism of the learning process, such as the function of dopaminergic neurons and other circuits in reward prediction error and how the reward prediction error drives different learning processes and guides decision-making. Studies about the way of the reward prediction error signal generated by upstream neural circuits have made certain progress, while there is much more to do. Moreover, we must better understand how the reward prediction error regulate dopamine release with the complex axonal arbors of dopamine midbrain neurons. Different release mechanism could regulate dopamine release and to further lead into the diverse function of dopamine system. Considering the different neural mechanism and its complicated interacting net, it may take a long time to deliberate. Besides, it is prospective to have better understand about the impact of gender, development and disease in the mechanism above. Therefore, future studies would be required to investigate the mechanisms of neuronal circuits across a wide range of learning processes. In general, this work helps better understand the association between reward prediction error signal and learning-related processes in different aspects of neural encoding, behaviors and diseases.

## Author contributions

YD: conceptualization, formal analysis, investigation, writing–original draft, and writing–review and editing. DS: conceptualization, investigation, validation, and writing–review and editing. JN: conceptualization and validation. HQ: conceptualization, validation, project administration, and supervision. ZQ: validation, project administration, writing–review and editing, and supervision. All authors contributed to the article and approved the submitted version.

## Funding

This research was supported by the National Science and Technology major projects (STI2030-Major Projects 2022ZD02068000), the National Natural Science Foundation of China (Grant Nos. 92049102, 32070954), Beijing Nova Program (Grant No. 20220484083) and Beijing Municipal Natural Science Foundation (Grant No. 7222113).

## Conflict of interest

The authors declare that the research was conducted in the absence of any commercial or financial relationships that could be construed as a potential conflict of interest.

## Publisher’s note

All claims expressed in this article are solely those of the authors and do not necessarily represent those of their affiliated organizations, or those of the publisher, the editors and the reviewers. Any product that may be evaluated in this article, or claim that may be made by its manufacturer, is not guaranteed or endorsed by the publisher.
